# A robust empirical seasonal prediction of winter NAO and surface climate

**DOI:** 10.1038/s41598-017-00353-y

**Published:** 2017-03-21

**Authors:** L. Wang, M. Ting, P. J. Kushner

**Affiliations:** 1 Lamont-Doherty Earth Observatory, Columbia University, Palisades, New York USA; 2 Department of Physics, University of Toronto, Toronto, Ontario Canada

## Abstract

A key determinant of winter weather and climate in Europe and North America is the North Atlantic Oscillation (NAO), the dominant mode of atmospheric variability in the Atlantic domain. Skilful seasonal forecasting of the surface climate in both Europe and North America is reflected largely in how accurately models can predict the NAO. Most dynamical models, however, have limited skill in seasonal forecasts of the winter NAO. A new empirical model is proposed for the seasonal forecast of the winter NAO that exhibits higher skill than current dynamical models. The empirical model provides robust and skilful prediction of the December-January-February (DJF) mean NAO index using a multiple linear regression (MLR) technique with autumn conditions of sea-ice concentration, stratospheric circulation, and sea-surface temperature. The predictability is, for the most part, derived from the relatively long persistence of sea ice in the autumn. The lower stratospheric circulation and sea-surface temperature appear to play more indirect roles through a series of feedbacks among systems driving NAO evolution. This MLR model also provides skilful seasonal outlooks of winter surface temperature and precipitation over many regions of Eurasia and eastern North America.

## Introduction

The North Atlantic Oscillation (NAO), the regional manifestation of the Arctic Oscillation (AO)^1^, is the leading mode of atmospheric variability and shows a strong link to weather and climate in the North Atlantic sector, including North America and Europe. The winter seasonal forecast of extreme climate anomalies, including extreme temperatures, heavy snowfall, droughts, and floods in North America and Europe, is reflected strongly in how well one can predict the phases of NAO^2, 3^. For example, the extremely negative phase of the NAO during the winter of 2009/2010 was associated with an exceptionally cold winter in Europe^2^ as well as heavy snowfall and record-breaking cold temperature across the east coast of North America^3^ that were life threatening and led to very heavy energy demand^4^.

The NAO varies on time scales ranging from daily to seasonal to decadal. On daily time scales, NAO variability is largely related to internal atmospheric dynamics involving fluctuations of the intensity and position of the North Atlantic westerly jet stream^5^. On monthly to seasonal time scales, the NAO variability is additionally influenced by fluctuations in boundary fields external to the atmosphere, such as sea-surface temperatures (SST) and sea ice concentrations (SIC)^6–9^. On decadal and longer time scales, the NAO is further influenced by natural and anthropogenic radiative forcing such as those associated with greenhouse gas increases^10^. Moreover, the NAO dynamics also involves multiple spatial scales. For example, both synoptic scale eddies^1^ and planetary scale waves^11^ are considered major sources of NAO variability.

Although numerous efforts have been made to improve forecast skill of NAO, it has remained largely unpredictable over seasonal and longer time scales. It is an open question what level of predictability can be reached beyond the limited skill currently achieved for both dynamical and statistical approaches. For dynamical models, state-of-the-art forecast skill (as measured by the anomaly correlation coefficient, ACC) of DJF-mean NAO has just recently increased to a significant level of 0.62 for 1993–2012 by the United Kingdom Meteorological Office (UKMO) Global Seasonal forecast System 5 (GloSea5)^12^ and for 1981–2016 by the UKMO Decadal Climate Prediction System 3 (DePreSys3)^13^ initialized on November 1st of each year. This high level of skill, which is accompanied by skilful prediction of the NAO one year ahead in DePreSys3, appears to require a large number of ensemble members (24 for GloSea5 and 40 for DePreSys3). Other operational forecast models usually show much poorer skills (up to 0.45)^14^, although some of them might gain extra skill if ensemble members are increased. For statistical models, a recent benchmark in wintertime NAO prediction is an ACC of 0.60 for 1981–2016 using November information^13^.

The slowly varying factors influencing seasonal-to-interannual variability in the winter NAO primarily include SST, SIC, El Niño/Southern Oscillation (ENSO), and the quasi-biennial oscillation (QBO)^12–15^. The Atlantic SST has classically been recognized as a useful predictor for the winter NAO^6, 7, 16^. Summer and fall SST anomalies from the extra-tropical and the equatorial Atlantic are found to correlate strongly with subsequent winter NAO anomalies. Moreover, summertime (May through September) Arctic sea ice concentration and extent have gained attention in recent years for their close connections with the winter AO/NAO^17–22^. However, Atlantic SST or Arctic sea ice alone are not sufficient to provide skilful statistical prediction of the winter NAO^22, 23^. Efforts to combine some of these predictors have exhibited slightly higher, yet still limited, skill than individual predictors^13^. Here we ask whether additional predictors could be found to improve on past efforts to predict the winter NAO with statistical approaches.

The winter NAO/AO has also been linked to stratospheric polar vortex variability^24, 25^, through downward coupling in zonal mean circulation^26^ or planetary waves^27^. Stratospheric polar vortex variability has been linked to extra-tropical tropospheric variability^28^ and tropical modes including ENSO and QBO^29, 30^. For example, the impacts of Arctic sea ice and Eurasian snow extent in shaping the NAO/AO are mediated through troposphere-stratosphere coupling^21, 31–34^. Similarly, ENSO modulates the polar vortex by inducing a PNA-like pattern that modifies the extra-tropical tropospheric wavenumber-1 and hence the upward wave activity^29, 30^. On the other hand, a strong polar night jet in the NH often occurs in the westerly phase of the QBO^29, 35^ (westerly at 10–50 hPa in the tropics). QBO affects the extra-tropical upward wave activity by changing the stratospheric zonal mean wind field, which in turn modulates the polar vortex^31, 36^. While ENSO and QBO impacts on the winter NAO are principally realized through their modulation of the polar vortex, it appears that ENSO and QBO impacts might potentially interfere with each other^29^. Therefore, their net impact might be better represented by the corresponding signature in extra-tropical stratospheric variability, which could provide a new predictor for statistical forecast of the winter NAO.

## Results

### Empirical model skill of winter NAO

**Figure 1:**
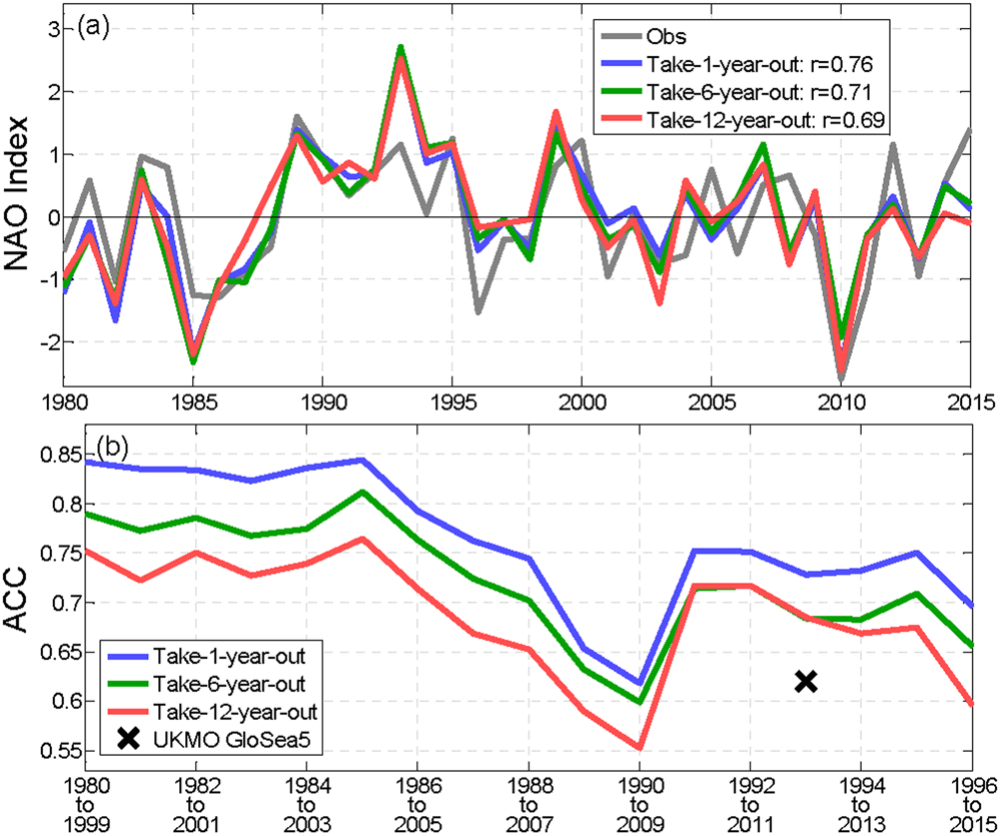
(**a**) The observed and predicted DJF-mean NAO index for the 1980–2015 period. (**b**) The forecast skill of each 20-year window. NAO is defined as the difference between the monthly mean sea level pressure (SLP) anomalies averaged over the domains of (50°W–10°E, 25–55°N) and (40°W–20°E, 55–85°N). Similar results are obtained with several other popular NAO indices (see Methods). The year corresponds to the January of each DJF season.

An empirical NAO forecast model is constructed here by including selected information about SST, SIC, and the stratospheric polar vortex, using the MLR technique (see Methods) similar to ref. 13. The MLR model predictions of the DJF-mean NAO (Fig. 1a) are based on selected Principal Components (PCs) of SST, SIC, and 70 hPa geopotential height (Z70 hPa), by the take-1/6/12-year-out cross-validation (out-of-sample) method. Only September and October PCs are included in order to fairly compare with dynamical forecasts initialized on November 1st of each year, whereas the statistical forecasts in ref. 13 used exclusively November predictors. In addition to the common take-1-year-out cross- validation, more stringent take-6-year-out (6-fold) and take-12-year-out (3-fold) cross- validations are adopted to examine the robustness of the model. The skill of these forecasts as measured by ACC ranges from 0.69 (take-12-year-out) to 0.76 (take-1-year-out), all significant at the 0.1% level by Student’s *t*-test and higher than the UKMO DePreSys3 and its corresponding statistical predictions^13^. The extremely negative NAO winter of 2009/2010 was well captured by the forecast in all three cases with almost perfect amplitude (Fig. 1a), while the UKMO GloSea5 and DePreSys3 ensemble means underestimated the amplitude of the 2010 extreme negative NAO (Fig. 1 in refs 12 and 13). Nevertheless, the MLR model overestimates 1985 and 1993 amplitudes and underestimates 2012 and 2015 amplitudes, which is partially due to the nonlinear trends in SIC (see Methods and Supplementary Fig. S4a).

To examine further whether the forecast skill is sensitive to different time periods, the anomaly correlations for 20-year moving windows, e.g., from 1979/80 to 1998/99, or 1995/96 to 2014/15, selected from the 36-year period were constructed (Fig. 1b). The anomaly correlation fluctuated within the range of approximately 0.55 to 0.85 for the varying 20-year periods, with the lowest forecast skill for the period 1990–2009 during which the NAO is relatively inactive. The forecast skill is competitive with or greater than state-of-the-art dynamical models (e.g., the cross symbol in Fig. 1b, which represents the skill of the 24-member ensemble mean hindcast by the UKMO GloSea5, one of the best dynamical models in NAO seasonal predictions^14^).

**Figure 2:**
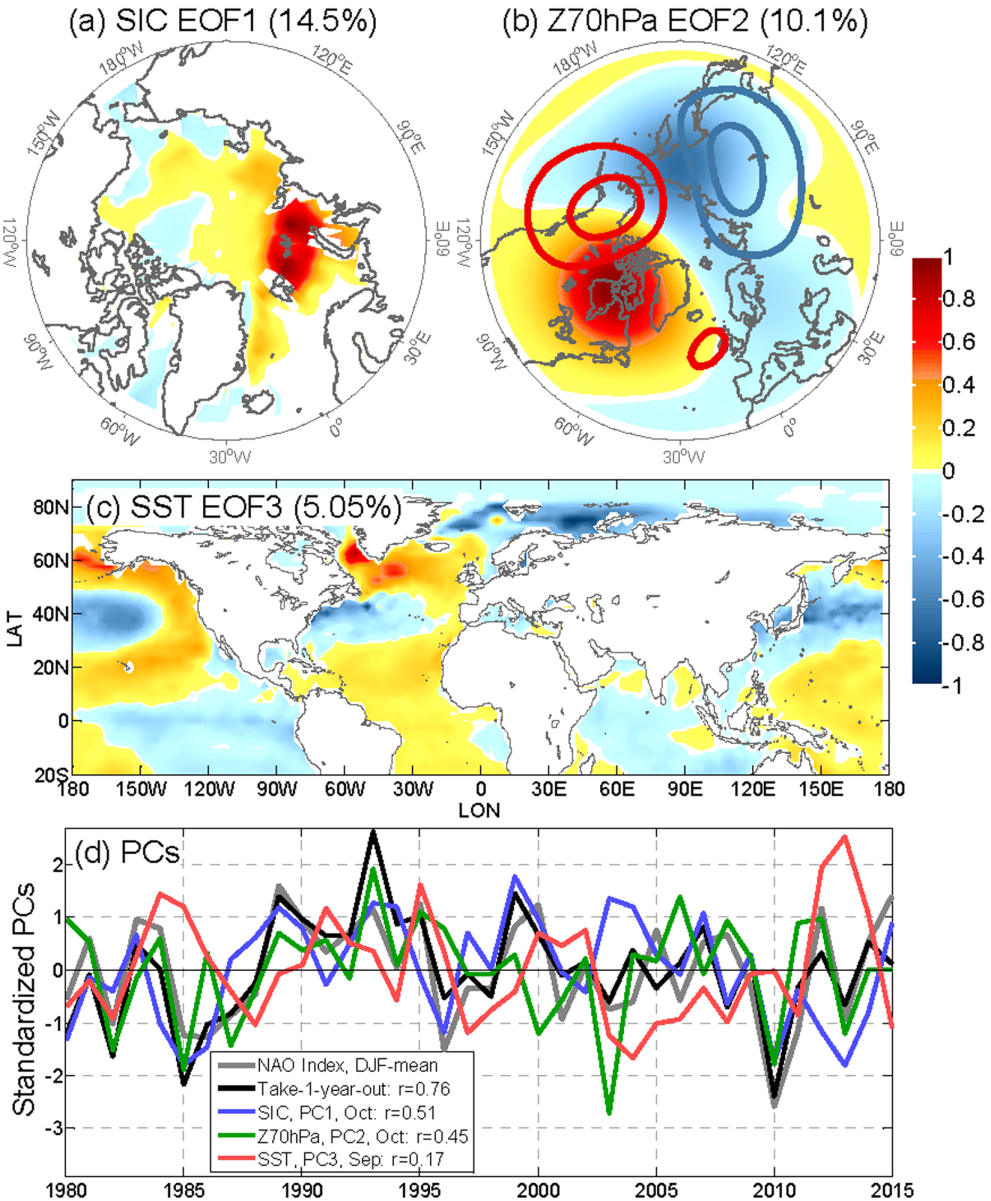
EOF patterns (in normalized units) of (**a**) the first mode of SIC, (**b**) the second mode of Z70 hPa, and (**c**) the third mode of SST, for months from September to February (SONDJF). (**d**) Standardized PCs compared with the observed and predicted DJF-mean NAO index (the numbers represent their ACCs). The numbers in the titles of (**a–c**) represent the portion of the variance explained by each mode. The climatological mean zonally asymmetric geopotential height is plotted in (**b**) as contours with 1000 m interval (positive/negative values in red/blue, respectively). The maps were generated by MathWorks MATLAB R2013b with M_Map (http://www.eos.ubc.ca/~rich/map.html).

The three MLR predictors are selected to optimize forecast skill^37^ from 18 potential candidate predictors: the September and October PCs of the first three Empirical Orthogonal Function (EOF) modes of monthly mean SST, SIC, and Z70 hPa (see Methods). The EOF modes were constructed from fall-winter season (SONDJF) monthly time series of 1979–2015 SIC, Z70 hPa, and SST, while the PCs were calculated by regressing September or October data on these SONDJF EOF patterns. These EOF modes illustrate the dominant patterns of Arctic sea ice, the lower stratosphere polar vortex, and the SST variability (Supplementary Fig. S1). The optimal predictors in the NAO MLR model are the leading mode of SIC (Fig. 2a), the second mode of Z70 hPa (Fig. 2b), and the third mode of SST (Fig. 2c). The leading mode of SIC features patterns localized in the Barents and Kara Seas (BKS) during the freezing season (September to January), similar to the pattern and location of the SIC anomaly in previous studies^18, 23^ (note that ref. 13 used sea ice extent in this region as a predictor). The second mode of Z70 hPa has a dipolar pattern over eastern Siberia and northern Canada (Fig. 2b) and its positive phase is characterized by an eastward shift of the climatological stationary wave (contours in Fig. 2b) or, in other words, a distortion in the polar vortex. This pattern is similar to that of the standing component of the temporal anomaly of planetary waves^38^, and might be linked to November zonal mean zonal winds (one of the predictors in ref. 13) through wave-mean flow interactions. The third mode of SST (SST EOF3) shows a tri-polar pattern in the Atlantic sector (Fig. 2c; a warm centre in mid-latitudes with a cold anomaly on the tropical and polar side, respectively) similar to those previously identified^13, 16, 39^. This SST pattern also has significant projections in the Norwegian Sea, the BKS, and the Laptev Sea, as well as the Pacific.

### Sources of NAO predictability

In this MLR model, the SIC is the most important source of predictability among the three predictors, as indicated by their temporal correlations with the DJF-mean NAO index (Fig. 2d). The SIC explains nearly half of the prediction skill (Supplementary Table S2b). The Z70 hPa and SST contribute comparably to the remaining half of the forecast skill (Supplementary Table S2b). This is consistent with ref. 12’s finding that Kara Sea ice dominates the source of NAO predictability in UKMO GloSea5. The Atlantic Ocean heat content, ENSO, and QBO are also identified as important sources by ref. 12. In our MLR model, Atlantic SST information could serve as a proxy for Atlantic Ocean heat content, and the combined effects of ENSO and QBO might be represented by Z70 hPa EOF2 (because their impacts on NAO are difficult to isolate). Therefore, this MLR model uses these three predictors to represent most key sources of predictability identified by the dynamical models. These predictors might also include potential sources that have not been previously identified such as the tri-band SST pattern in the Pacific sector (Fig. 2c).

**Figure 3:**
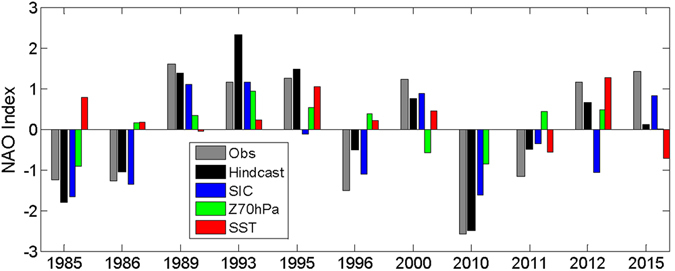
Contribution of NAO hindcast (grey) from October SIC PC1 (blue), October Z70 hPa PC2 (green), and September SST PC3 (red), for strong NAO winters (amplitude > 1.1).

The empirical forecast model suggests several dynamical mechanisms acting in driving interannual variability in the NAO. It has been proposed that a positive anomaly in the BKS SIC in the fall could cool the atmosphere in the Atlantic sector and induce a strengthening and poleward shift of the extra-tropical jet, resulting in the positive phase of NAO in the winter and vice versa^20, 32, 33^. This mechanism has also been used to explain the atmospheric circulation response to projected Arctic sea ice loss^19, 21^. The relatively high positive correlation (0.51) between PC1 of SIC in October and the following winter’s NAO confirms these results. In particular, this predictor is the dominant factor for both the extreme negative NAO winter in 2010 and the extreme positive NAO winter in 1989, as well as in 1985, 1986, and 1996 when NAO amplitude was large (Fig. 3; see also Supplementary Table S2a).

The tri-polar SST pattern in the Atlantic sector has long been believed to be able to induce a NAO-like anomaly in the atmospheric circulation, directly through diabatic heating and indirectly through transient eddy-mean flow interactions^40^. However, this tri-polar pattern also resembles those of the winter-spring SST anomalies induced by the winter NAO anomalies^7^. This indicates that the tri-polar SST pattern could be a result of an atmospheric- (i.e., winter NAO-) forced anomaly that penetrates deeply into the winter mix layer of the Atlantic Ocean, persists through spring and summer, and re-emerges at the sea surface in the fall^39, 41^. Thus a possible coupling between the ocean and atmosphere could be linked to NAO predictability in the MLR model. In addition, the Pacific SST also shows a similar tri-polar pattern at the same latitude bands as those in the Atlantic sector. These synchronized patterns imply that this SST mode reflects the part of the meridional variability of the position of the tropospheric jet and storm track that is somewhat linked between the two ocean basins, possibly through the Northern Annular Mode^42^. Furthermore, the relatively strong amplitude in the Norwegian Sea in this SST mode also reflects the interaction between this mode and the sea ice variability there. This predictor was in near-neutral phases in the most extreme NAO winters of 1989 and 2010 but played a large role in 1995, 2000, and 2015 winters (Fig. 3; see also Supplementary Table S2a).

**Figure 4:**
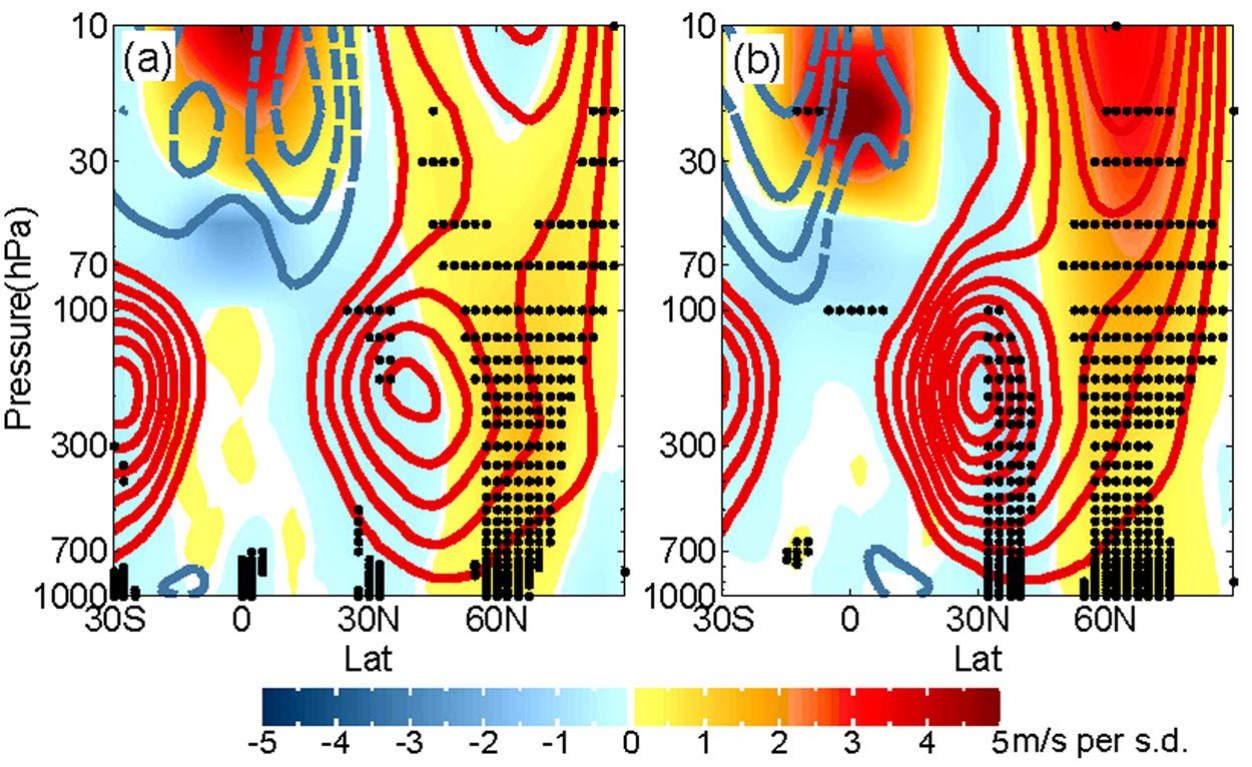
(**a**) October and (**b**) DJF-mean zonal mean zonal wind regressed onto the October Z70 hPa PC2 (shaded; in m/s per standard deviation of this PC), compared with climatological mean states (contours with an interval of 5 m/s; positive/negative values in red/blue, respectively). The black dots indicate the 95% confidence interval of the regression based on a two-tailed *t*-test.

The Z70 hPa EOF2 does not seem to make leading contributions to the winter NAO in individual years except for 1983 when all three predictors lead to negative winter NAO (Supplementary Table S2a), but it often amplifies the sea ice effect when SIC EOF1 dominants (e.g., in 1985, 1989, 1996, and 2010 winters; see Fig. 3 and Supplementary Table S2a). To explore the connection between the second mode of Z70 hPa and the winter NAO, the zonal-mean zonal wind is regressed onto the October PC time series, which shows a strengthening and poleward shift of the tropospheric extra-tropical jet in October associated with the positive phase of the Z70 hPa EOF2 (Fig. 4a). This anomaly in the extra-tropical jet persists well into winter (Fig. 4b; see also Supplementary Fig. S5), which is largely manifested in the Atlantic sector (Supplementary Fig. S6) and thus induces a positive phase of the NAO.

This linkage between the poleward (equatorward) shift and strengthening (weakening) of the extra-tropical jet and the positive (negative) phase of winter NAO is consistent with previous studies^5, 24, 43^, and thus this Z70 hPa mode can serve as a stratospheric predictor for the winter NAO. In addition, a positive anomaly in the winter polar vortex is also found to often accompany a positive NAO^24, 26^. The significant connection between the October Z70 hPa EOF2 and the winter polar night jet (Fig. 4b and Supplementary Fig. S5) further supports that this mode is a good indicator of the winter NAO. Furthermore, if November predictors are allowed in the model selection, the best stratospheric predictor becomes Z70 hPa EOF1, which is approximately zonally symmetric and therefore dynamically similar to the stratospheric predictor of November zonal mean zonal wind averaged over 60–80°N in ref. 13. Note that the other two predictors do not induce this combination of a strengthening and poleward shift of the extra-tropical jet persisting from October into winter, although the October SIC EOF1 does induce a similar change in the tropospheric/stratospheric jet in DJF (Supplementary Fig. S7). This indicates that it takes a couple of months for the atmospheric response to sea ice anomalies to propagate from the surface to the stratosphere and come back down to the surface to influence the NAO^32, 33^. The regressed zonal mean zonal wind also shows a westerly QBO like pattern in the tropics, indicating that this Z70 hPa mode is consistent with expected QBO impacts on the polar vortex and hence the NAO^29^.

The Snow Advance Index (SAI), which is a measure of the rate of the advance of the snowline of Eurasia south of 60°N in the month of October, has been widely used for seasonal forecasts of winter AO^34^, although the robustness of this relationship outside the satellite era has been questioned^44^. The SAI has a significant correlation (0.46) with October Z70 hPa PC2 for 1979–2014. This is dynamically consistent as October Eurasian snow is largely related to the stratospheric variability through upward wave propagation^34^. The Z70 hPa PC2 can also induce changes in the tropospheric and stratospheric jets (Supplementary Figs S5 and S7) that are similarly correlated with the SAI^34^. However, the NAO forecast skill slightly degrades when Z70 hPa PC2 is replaced by the SAI in the MLR model (Supplementary Fig. S8), indicating that Z70 hPa PC2 might better represent the stratospheric source of NAO predictability.

### Empirical model skill of winter surface climate

**Figure 5:**
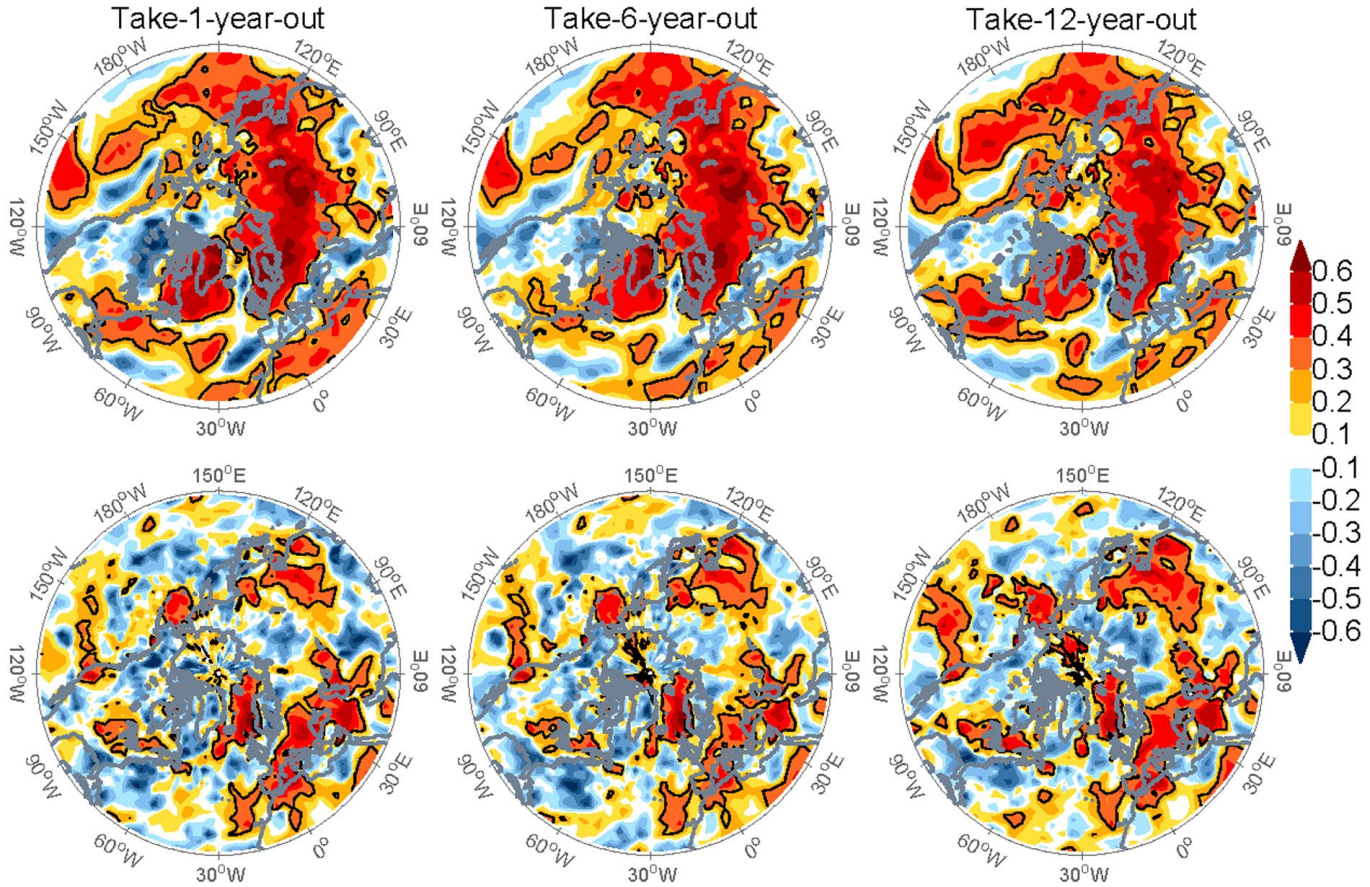
The forecast skill (ACC) of DJF-mean 2 m air temperature (upper row) and GPCP precipitation (lower row) by take-1(left)/6(middle)/12(right)-year-out cross-validation. The black contours indicate the ACC is statistically significant at 95% confidence interval based on a one-tailed *t*-test (as only positive ACC is considered skilful). The maps were generated by MathWorks MATLAB R2013b with M_Map (http://www.eos.ubc.ca/~rich/map.html).

This MLR model can also be applied to the seasonal outlook of winter surface climate, given the close connection between the winter surface climate and NAO. The surface temperature from the European Centre for Medium-Range Weather Forecasts ERA-Interim reanalysis^45^ and precipitation from the Global Precipitation Climatology Project (GPCP)^46^ are predicted on each grid point. The cross validated skill of DJF-mean 2 m air temperature is primarily significant over the Eastern United States and Canada, Greenland, and most of Eurasia (Fig. 4a–c). The precipitation is primarily well predicted over Mediterranean regions and East Asia, as well as parts of Eastern United States and Northern Europe. The above forecast skills are quite robust as shown by the spatial coherence and consistency in the take-1/6/12-year-out cross-validations (Fig. 5). The ability of this MLR model to predict both the winter NAO index and surface climate in the relevant regions indicates that the model and the predictors represent the underlying physical processes associated with persistent fall-to-winter seasonal variability in the Northern Hemisphere.

## Discussion

In summary, the predictive information from the sea ice field, the lower stratospheric circulation, and ocean surface temperatures has been combined to produce a seasonal forecast of the winter NAO using a statistical model. The simple MLR model is able to provide skilful predictions of the DJF-mean NAO index and surface climate using only September and October initial conditions, with NAO forecast skill comparable to or even higher than that of state-of-the-art dynamical models. This statistical NAO prediction model can further improve the seasonal outlook of the winter surface conditions in the pan-Atlantic region.

NAO seasonal variability is controlled by coupled nonlinear processes such as wave-mean flow interactions and atmosphere-ocean interactions^1, 40^. It is intriguing that such a highly coupled system can be well represented by a simple linear model consisting of three predictors, at least as effectively as many current dynamical prediction systems^12–14^. The existence and effectiveness of these predictors for the winter NAO suggests promising directions for exploring mechanisms of, for example, how ENSO and QBO influences the extra-tropical lower stratospheric circulation. This statistical model can also be used as a useful diagnostic tool to determine the NAO predictability, or lack thereof, in the coupled ocean-atmosphere models, such as those used for the Coupled Model Intercomparisons Project Phase 5 (CMIP5) and future generations of these models. These seasonal prediction systems are often too noisy and their extratropical signals too weak^12, 13, 15^, suggesting possible unrealistic NAO dynamics. Reliable future projections of the climate depend heavily on how well the physical linkages between the different components of the climate system are represented in these models. Thus the models’ ability to represent the statistical linkages between NAO and other physical processes as shown in observations could produce useful metrics for model validation.

## Methods

### Data

The monthly-mean SLP is used to define the NAO index and used as the predictand, and the leading PCs of Z70hPa, SIC, and SST are used as predictors. The data used are 1979 to 2015 monthly fields from ERA-Interim reanalysis at 2.5° × 2.5° resolution. The long term trend and climatological annual cycle are removed from all variables at each grid point before the EOF analysis. Three additional NAO indices are used to test the robustness of our model to the definition of the NAO index, including the station-based definition^47, 48^ and the PC-based definition^49, 50^ using SLP anomalies, as well as the Rotated Principal Component-based definition^51^ using 500 hPa geopotential height anomalies by the Climate Prediction Center. The monthly-mean ERA-Interim 2 m air temperature and GPCP precipitation are also used as predictands to examine their seasonal forecast skill.

The NAO index is defined here as the difference between the SLP anomalies averaged over the domains of (50°W–10°E, 25–55°N) and (40°W–20°E, 55–85°N) (grey line in Fig. 1a; see also in Supplementary Fig. S3a and referred as the “ERA-Interim Domain-based” definition). The domains are chosen based on the nodal positions of the EOF patterns^49^, i.e., the empirical positions of the Azores Subtropical High and the Icelandic Subpolar Low. This definition conforms to both the station-based definition^47^ and the PC-based definition^49^, with correlation coefficients of greater than 0.9 for the 1980–2015 DJF-mean indices with different definitions. The results shown here are fairly insensitive to the precise location and size of the domains (not shown), as well as different definitions of the NAO index (Fig. 1a and Supplementary Fig. S3b–d).

The EOF analysis is performed using Z70 hPa, SIC, and SST from September through February (SONDJF) of each year for 1979–2015 (Supplementary Fig. S1). Only the first three PCs from each variable are considered as potential predictors to ensure the robustness of the cross-validation. The domain north of 20°N is chosen for EOF decomposition for Z70 hPa and SIC to focus on the extra-tropical variability, whereas the area north of 20°S is chosen for SST to also include key regions of tropical SST variability.

### Multiple Linear Regression (MLR) model and cross-validation

Here we construct an empirical model for NAO prediction using the MLR technique^13, 37, 52^. The NAO index is represented by the linear combination of several predictors. We used the take-*N*-year-out cross-validation (*N* = 1, 6, or 12) to validate the MLR prediction model. For the example of *N* = 12, the 36 years of data (1979–2015) are first divided into 3 consecutive 12-year segments. Each time a 12-year segment (one third of the entire period) is excluded in the EOF decomposition and the September-October conditions of this 12-year period are only used to generate the initial conditions for the forecast. The MLR model is trained with the remaining 24 years of data and used to predict the DJF mean NAO index of the excluded 12-year period. This procedure is repeated for all three 12-year segments to obtain the take-12-year-out cross-validated prediction of the NAO index. This cross-validation procedure guarantees that the prediction-period data are strictly excluded in the training process.

### Model selection

Although we have narrowed down the potential predictors to the September and October PCs of the three leading modes of three variables, i.e., 2 × 3 × 3 = 18 in total (Supplementary Table S1a), it is necessary to search for the optimal set of predictors, given the limited length of available observations (especially SIC). A simple and quick way to do so is the forward stepwise selection^37^: the predictors are added to the MLR model one at a time. At the first step, each of the 18 PCs is used alone to calculate the forecast skill (as measured by the ACC) for the winter NAO and the best predictor, the October SIC PC1 (P03 in Supplementary Table S1a), is retained in the MLR model. At the second step, each of the remaining 17 PCs is added to predict the winter NAO along with P03 and the October Z70hPa PC2 (P04 in Supplementary Table S1a) is found to improve the ACC the most. The above procedure is repeated until all 18 PCs are included in the MLR model. The hindcast skill increases rapidly from the single predictor case to the three-predictor case and then increases slowly (circles in Supplementary Fig. S2), while the take-1-year-out cross-validation skill peaks at the three-predictor case (squares in Supplementary Fig. S2). Although the take-6-year-out and take-12-year-out cross-validation skills peak with seven predictors, the improvements in the ACC are rather small relative to the three-predictor case (diamonds and triangles in Supplementary Fig. S2). Therefore, the optimal number of predictors is chosen as 3. The all-possible-regressions procedure^37^ confirms that the best set of predictors consists of the following three: October PC 1 of the SIC, October PC 2 of the 70hPa geopotential height, and the September PC 3 of the SST (P03, P04, and P17 in Supplementary Table S1a). The three predictors identified here are just one of many possible sets of predictors that can describe the winter NAO from the complex atmosphere-ocean-cryosphere system. For example, the second best set also includes the first two predictors but the third one is the October PC 3 of the SST (P08 in Supplementary Table S1a), with a decrease in ACC of about 0.04. We do not intend to exhaust all possibilities here (i.e., explore other possible variables) but rather provide an example of feasible seasonal prediction of the winter NAO.

### Contributions of individual predictors

The contribution of each predictor is estimated as the reduction in ACC due to removing this predictor from the MLR model (while keeping the other two predictors). This estimation can avoid influences from possible collinearity among predictors. The existence of collinearity generally does not jeopardize the MLR model’s ability in prediction but rather makes it difficult to properly attribute the impacts from involved predictors^37^. Our approach maximizes the contribution from the remaining predictors and shows the least impact of excluding individual predictors. The contribution is calculated for each of the three predictors and also for all three take-*N*-year-out cross-validation (*N* = 1, 6, or 12) cases (Supplementary Table S2b). The ACC reduces by nearly half when the October SIC PC 1 is excluded in the MLR model, and the other two predictors contribute much less.

Since the NAO is controlled by the highly coupled atmosphere-ocean-cryosphere system, redundancy is inevitable in the information from these predictors. For example, there exists weak collinearity (a correlation coefficient of −0.49; see also Supplementary Table S1b) between the SIC PC1 and SST PC3. The positive SIC anomaly in the north part of the Barents and Kara Seas (BKS) (Fig. 2a) are physically consistent with the negative SST anomaly (for the negative mode of SST EOF 3) in the south part of the BKS (Fig. 2c but with reversed sign), i.e., a lower SST is favourable for ice formation in the BKS. These two predictors share some information of the winter NAO and hence their impacts on the winter NAO are not separable explicitly. However, this weak collinearity does not seem to affect the robustness of the MLR model, as indicated by the high forecast skill of the take-12-year-out cross-validation.

### Sensitivity to detrending methods

In the manuscript we used the most common and traditional detrending method: a linear monthly trend is removed from the data at each grid point. This might not be the optimal way, for example, for the SIC which has shown much weaker trend before around 2000 than after^53^. This has potential negative impact on the model skill, such as overestimating the amplitude in the early years and underestimating in the late years (Fig. 1a and Supplementary Fig. S3b–d). Therefore, we tested the case of removing a third-order polynomial trend in the SIC (similar to ref. 23), as shown in Supplementary Fig. S4. There are small but visible improvements in Supplementary Fig. S4 compared with Fig. 1: 0.01 increases in ACC. More specifically, Supplementary Fig. S4a shows better match with observations in 1980–1982, 1993–1994, 1996, 2001, 2008, 2012, and 2015, albeit poorer match in 1995, 1997–1998, 2000, 2002, and 2013.

## Electronic supplementary material

Supplementary Tables and Figures for A robust empirical seasonal prediction of winter NAO and surface climate
