# Supplementary material for: A robust empirical seasonal prediction of winter NAO and surface climate
Source: Sci Rep. 2017 Mar 21;7:279. doi: 10.1038/s41598-017-00353-y (PMC5428292; doi:10.1038/s41598-017-00353-y)
Supplement: Supplementary file 1 — Supplementary Tables and Figures for A robust empirical seasonal prediction of winter NAO and surface climate [file 41598_2017_353_MOESM1_ESM.pdf]

Supplementary Tables and Figures for

A robust empirical seasonal prediction of winter NAO and surface climate

L. Wang[1], M. Ting[1], and P. J. Kushner[2]

[1]Lamont-Doherty Earth Observatory, Columbia University, Palisades, New York.

[2]Department of Physics, University of Toronto, Toronto, Ontario, Canada.

Supplementary Table S1a. List of potential predictors

	Variable	Mode	Month
P01	Z70hPa	PC1	October
P02	SST	PC1	October
P03	SIC	PC1	October
P04	Z70hPa	PC2	October
P05	SST	PC2	October
P06	SIC	PC2	October
P07	Z70hPa	PC3	October
P08	SST	PC3	October
P09	SIC	PC3	October
P10	Z70hPa	PC1	September
P11	SST	PC1	September
P12	SIC	PC1	September
P13	Z70hPa	PC2	September
P14	SST	PC2	September
P15	SIC	PC2	September
P16	Z70hPa	PC3	September
P17	SST	PC3	September
P18	SIC	PC3	September

The three predictors selected for the NAO prediction are highlighted.

Supplementary Table S1b. Cross correlation coefficients between potential predictors and DJF NAO index

	NAO	P01	P02	P03	P04	P05	P06	P07	P08	P09	P10	P11	P12	P13	P14	P15	P16	P17	P18
P01	0.20	1.00	0.15	-0.18	-0.40	0.29	0.15	0.41	0.53	-0.26	-0.08	0.14	-0.22	0.19	0.32	0.16	0.19	0.50	-0.28
P02	0.07	0.15	1.00	-0.19	-0.26	-0.10	-0.11	0.17	0.18	0.02	0.16	0.97	-0.24	0.07	-0.12	-0.15	0.33	0.20	-0.12
P03	**0.51**	-0.18	-0.19	1.00	-0.07	-0.15	0.20	-0.21	-0.54	0.59	-0.11	-0.15	0.83	-0.17	-0.18	0.17	-0.23	-0.49	0.68
P04	**0.45**	-0.40	-0.26	-0.07	1.00	0.08	0.14	-0.07	0.02	-0.08	0.10	-0.23	0.09	-0.33	-0.09	0.00	-0.42	-0.03	0.11
P05	0.05	0.29	-0.10	-0.15	0.08	1.00	0.11	-0.20	0.09	-0.16	-0.26	-0.01	-0.11	0.13	0.92	0.10	0.14	0.18	-0.08
P06	0.06	0.15	-0.11	0.20	0.14	0.11	1.00	-0.20	0.04	-0.15	0.23	-0.06	0.14	-0.35	0.13	0.89	-0.07	0.13	-0.11
P07	0.12	0.41	0.17	-0.21	-0.07	-0.20	-0.20	1.00	0.44	0.05	-0.04	0.13	-0.19	0.02	-0.16	-0.15	0.03	0.20	-0.11
P08	0.06	0.53	0.18	-0.54	0.02	0.09	0.04	0.44	1.00	-0.51	0.06	0.17	-0.59	0.23	0.12	-0.02	0.28	0.86	-0.57
P09	0.26	-0.26	0.02	0.59	-0.08	-0.16	-0.15	0.05	-0.51	1.00	0.13	0.02	0.47	-0.00	-0.18	-0.12	-0.15	-0.59	0.67
P10	0.14	-0.08	0.16	-0.11	0.10	-0.26	0.23	-0.04	0.06	0.13	1.00	0.17	-0.19	-0.26	-0.28	0.11	-0.21	0.11	-0.01
P11	0.10	0.14	0.97	-0.15	-0.23	-0.01	-0.06	0.13	0.17	0.02	0.17	1.00	-0.21	0.06	-0.04	-0.12	0.26	0.23	-0.09
P12	0.22	-0.22	-0.24	0.83	0.09	-0.11	0.14	-0.19	-0.59	0.47	-0.19	-0.21	1.00	-0.20	-0.10	0.08	-0.32	-0.53	0.86
P13	0.13	0.19	0.07	-0.17	-0.33	0.13	-0.35	0.02	0.23	-0.00	-0.26	0.06	-0.20	1.00	0.17	-0.38	0.27	0.13	-0.18
P14	0.04	0.32	-0.12	-0.18	-0.09	0.92	0.13	-0.16	0.12	-0.18	-0.28	-0.04	-0.10	0.17	1.00	0.18	0.25	0.19	-0.12
P15	0.11	0.16	-0.15	0.17	0.00	0.10	0.89	-0.15	-0.02	-0.12	0.11	-0.12	0.08	-0.38	0.18	1.00	-0.09	0.03	-0.21
P16	0.29	0.19	0.33	-0.23	-0.42	0.14	-0.07	0.03	0.28	-0.15	-0.21	0.26	-0.32	0.27	0.25	-0.09	1.00	0.37	-0.27
P17	0.17	0.50	0.20	**-0.49**	-0.03	0.18	0.13	0.20	0.86	-0.59	0.11	0.23	-0.53	0.13	0.19	0.03	0.37	1.00	-0.52
P18	0.12	-0.28	-0.12	0.68	0.11	-0.08	-0.11	-0.11	-0.57	0.67	-0.01	-0.09	0.86	-0.18	-0.12	-0.21	-0.27	-0.52	1.00

Predictors that have significant correlation with the NAO index are highlighted in green. The significant correlation among three selected predictors is highlighted in yellow.

Supplementary Table S1c. Forecast skill in ACC for the hindcast and take-*N*-year-out cross-validation in the forward stepwise selection

		Variable	Mode	Month	Hindcast	*N*=1	*N*=6	*N*=12
1	P03	SIC	PC1	October	0.51	0.47	0.47	0.48
2	P17	SST	PC3	September	0.70	0.64	0.60	0.58
3	P04	Z70hPa	PC2	October	0.80	0.76	0.71	0.69
4	P12	SIC	PC1	September	0.81	0.77	0.73	0.70
5	P16	Z70hPa	PC3	September	0.82	0.76	0.73	0.71
6	P06	SIC	PC2	October	0.83	0.74	0.73	0.71
7	P15	SIC	PC2	September	0.85	0.75	0.71	0.67
8	P07	Z70hPa	PC3	October	0.85	0.71	0.70	0.68
9	P09	SIC	PC3	October	0.86	0.72	0.73	0.69
10	P18	SIC	PC3	September	0.87	0.71	0.68	0.68
11	P08	SST	PC3	October	0.87	0.69	0.67	0.58
12	P10	Z70hPa	PC1	September	0.87	0.67	0.65	0.56
13	P05	SST	PC2	October	0.88	0.65	0.60	0.48
14	P14	SST	PC2	September	0.88	0.65	0.60	0.41
15	P01	Z70hPa	PC1	October	0.89	0.64	0.58	0.38
16	P11	SST	PC1	September	0.89	0.63	0.57	0.33
17	P02	SST	PC1	October	0.89	0.62	0.56	0.36
18	P13	Z70hPa	PC2	September	0.89	0.61	0.55	0.35

Supplementary Table S2a. Contributions of individual predictors in the hindcast

Year	NAO	Hindcast	$\alpha\times$SIC	$\beta\times$Z70hPa	$\gamma\times$SST
1980	-0.55	-1.22	-1.23	0.47	-0.45
1981	0.59	-0.02	-0.14	0.26	-0.14
1982	-1.03	-1.71	-0.37	-0.74	-0.60
1983	0.95	0.70	0.61	-0.08	0.17
1984	0.79	0.27	-0.95	0.29	0.93
1985	-1.25	-1.81	-1.66	-0.92	0.78
1986	-1.28	-1.04	-1.36	0.15	0.17
1987	-0.86	-0.78	0.18	-0.70	-0.26
1988	-0.47	-0.30	0.56	-0.19	-0.67
1989	1.59	1.38	1.10	0.34	-0.05
1990	0.93	0.97	0.73	0.18	0.07
1991	0.34	0.76	-0.27	0.27	0.75
1992	0.71	0.70	0.45	-0.08	0.33
1993	1.15	2.33	1.17	0.93	0.23
1994	0.05	0.78	1.11	0.05	-0.38
1995	1.25	1.46	-0.12	0.53	1.05
1996	-1.52	-0.50	-1.10	0.38	0.22
1997	-0.36	-0.17	0.65	-0.04	-0.78
1998	-0.34	-0.53	0.01	-0.03	-0.50
1999	0.81	1.49	1.62	0.14	-0.27
2000	1.22	0.75	0.89	-0.59	0.45
2001	-0.94	-0.03	-0.04	-0.28	0.30
2002	0.08	0.21	-0.39	0.11	0.49
2003	-0.74	-0.89	1.25	-1.33	-0.81
2004	-0.62	0.15	1.10	0.14	-1.09
2005	0.75	-0.31	0.28	0.08	-0.67
2006	-0.58	-0.03	-0.09	0.67	-0.61
2007	0.52	0.73	1.00	-0.04	-0.23
2008	0.65	-0.79	-0.60	0.45	-0.64
2009	-0.30	0.33	0.26	0.12	-0.05
2010	-2.58	-2.50	-1.61	-0.87	-0.01
2011	-1.15	-0.50	-0.36	0.43	-0.57
2012	1.15	0.67	-1.07	0.47	1.26
2013	-0.94	-0.61	-1.67	-0.59	1.65
2014	0.55	-0.06	-0.74	0.00	0.68
2015	1.41	0.11	0.83	0.01	-0.72

NAO Hindcast = $\alpha\times$SIC + $\beta\times$Z70hPa + $\gamma\times$SST, where $\alpha = 0.925$, $\beta = 0.486$, and $\gamma = 0.652$.

The most extreme NAO years are shaded in red (>2) and blue (<-2), with moderate NAO years in light red (1.1~2) and light blue (-2~-1.1).

Supplementary Table S2b. Contributions of individual predictors in the take-*N*-year-out cross-validation

	Variable	Mode	Month	*N*=1	*N*=6	*N*=12
P03	SIC	PC1	October	0.38	0.32	0.35
P04	Z70hPa	PC2	October	0.10	0.09	0.10
P17	SST	PC3	September	0.15	0.13	0.08

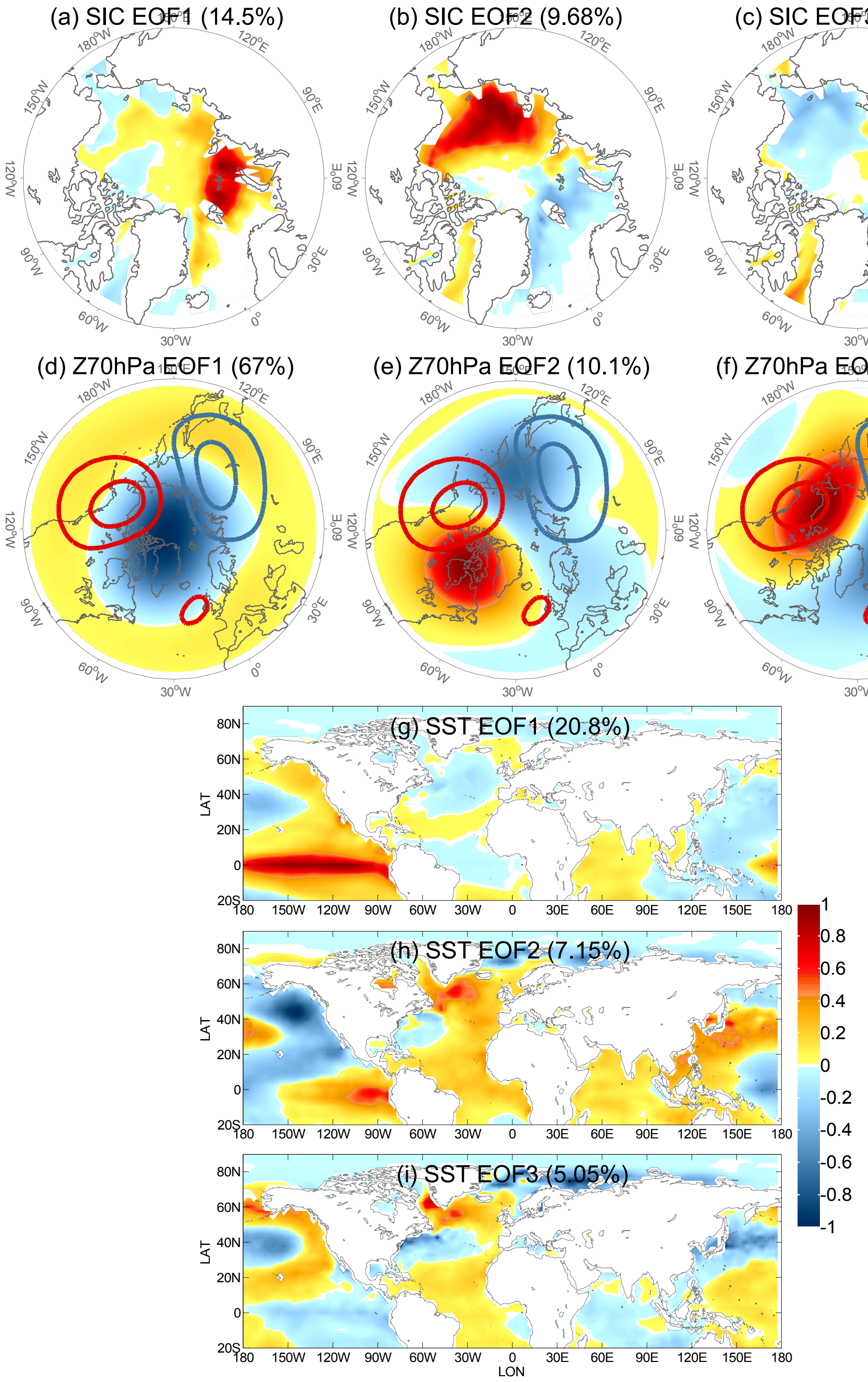

Supplementary Figure S1. Same as Fig. 2a-2c but for SONDJF EOF patterns (in normalized units) of the first three modes of SIC, Z70hPa, and SST. The maps were generated by MathWorks MATLAB R2013b with M_Map (http://www.eos.ubc.ca/~rich/map.html).

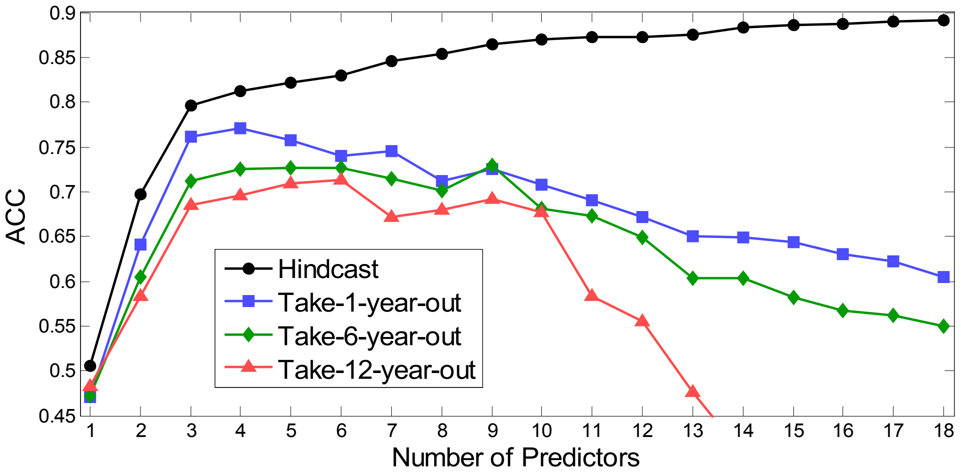

Supplementary Figure S2. Forecast skill (anomaly correlation coefficient) of the MLR model as a function of the number of predictors in the forward stepwise selection.

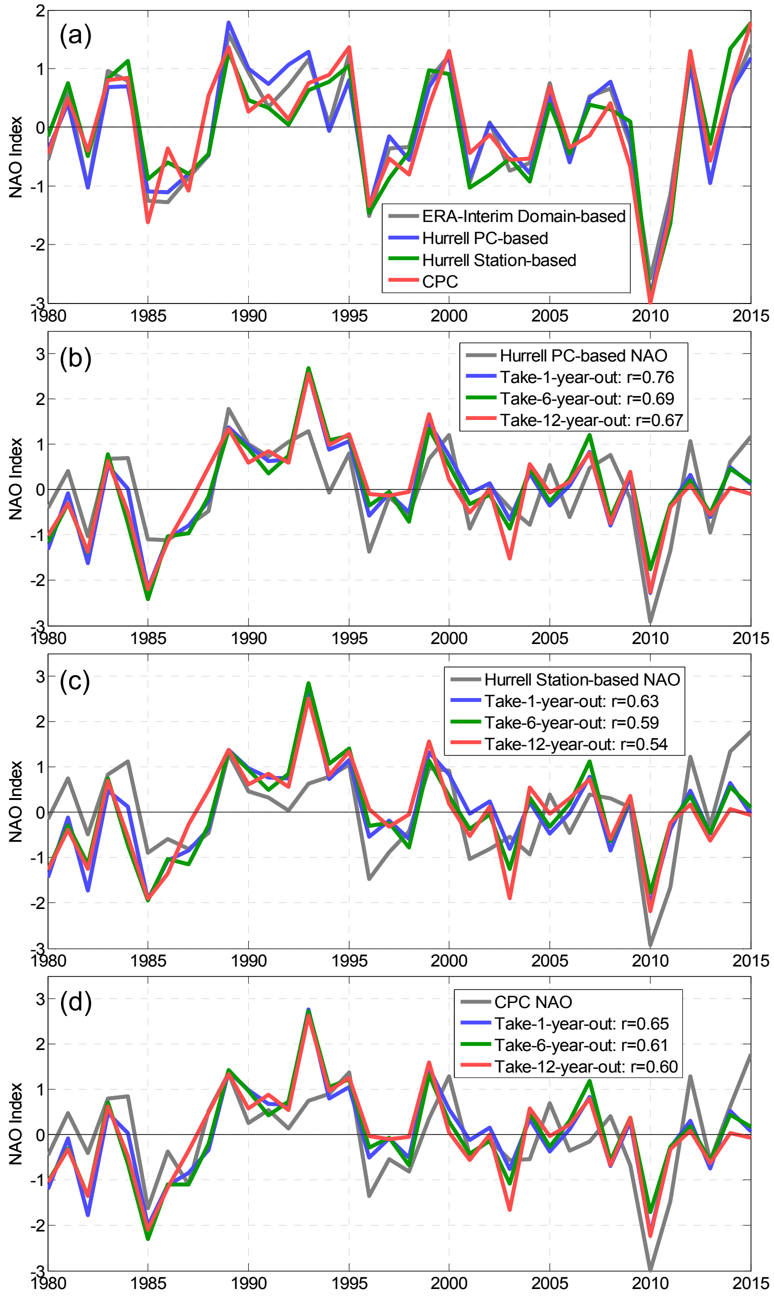

Supplementary Figure S3. (a) The DJF-mean NAO index for the 1980-2015 period with different definitions. (b) The Hurrell PC-based DJF-mean NAO index and its predictions for the 1980-2015 period. Same as (b) but for the (c) Hurrell Station-based and (d) CPC NAO definitions.

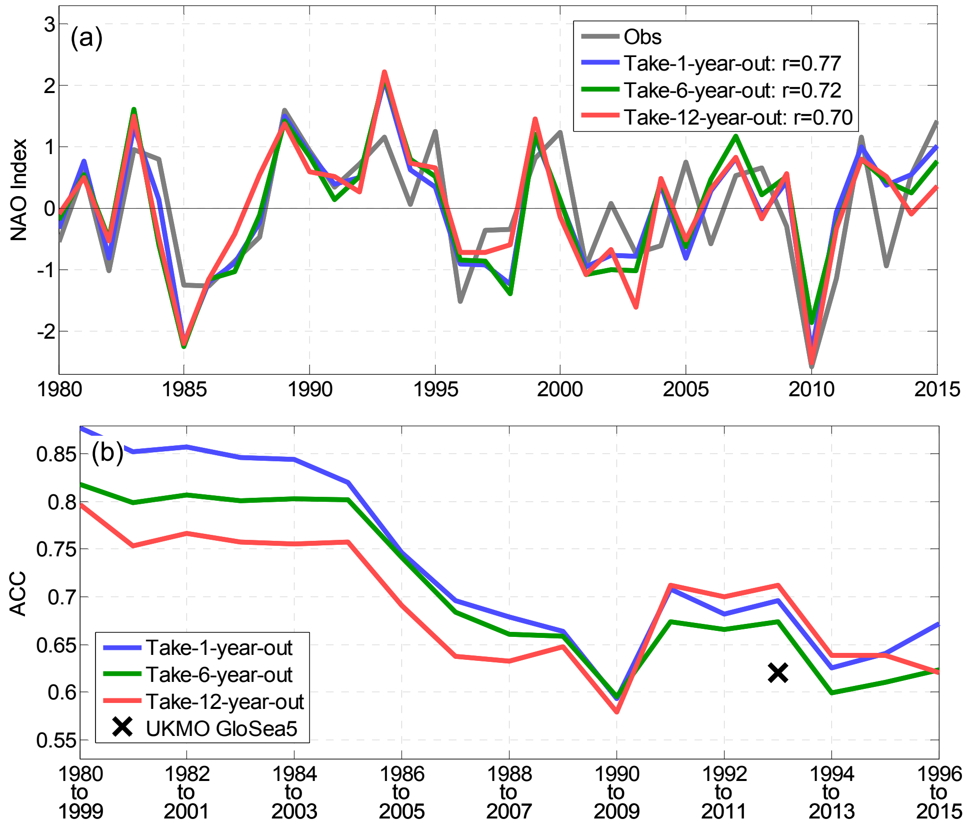

Supplementary Figure S4. Same as Fig. 1 but with a third-order polynomial trend (instead of a linear trend) removed from SIC at each grid point.

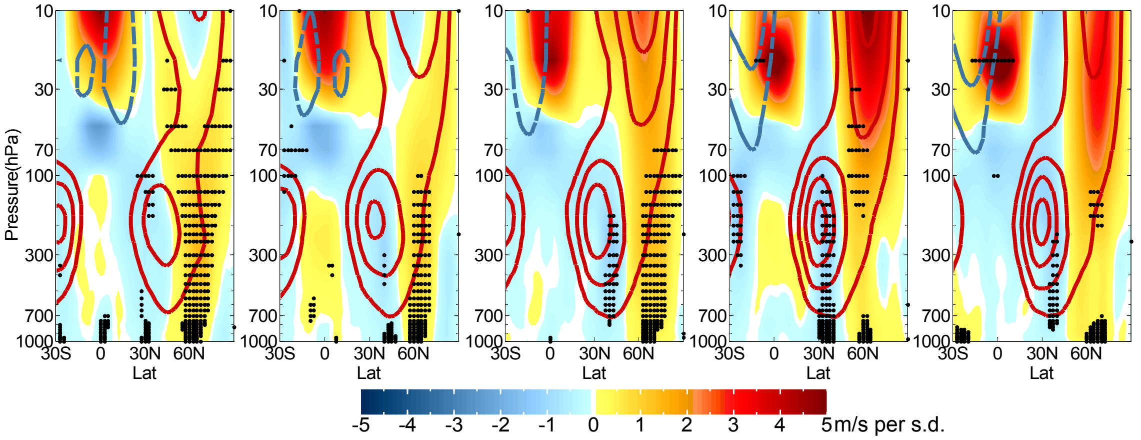

Supplementary Figure S5. October-December (left to right) zonal mean zonal wind regressed on the October Z70hPa PC 2 (shaded), compared with climatological mean states (contours with an interval of 10 m/s). The black dots indicate the 95% confidence interval of the regression based on a two-tailed *t*-test. The positive/negative values are shown in red/blue, respectively.

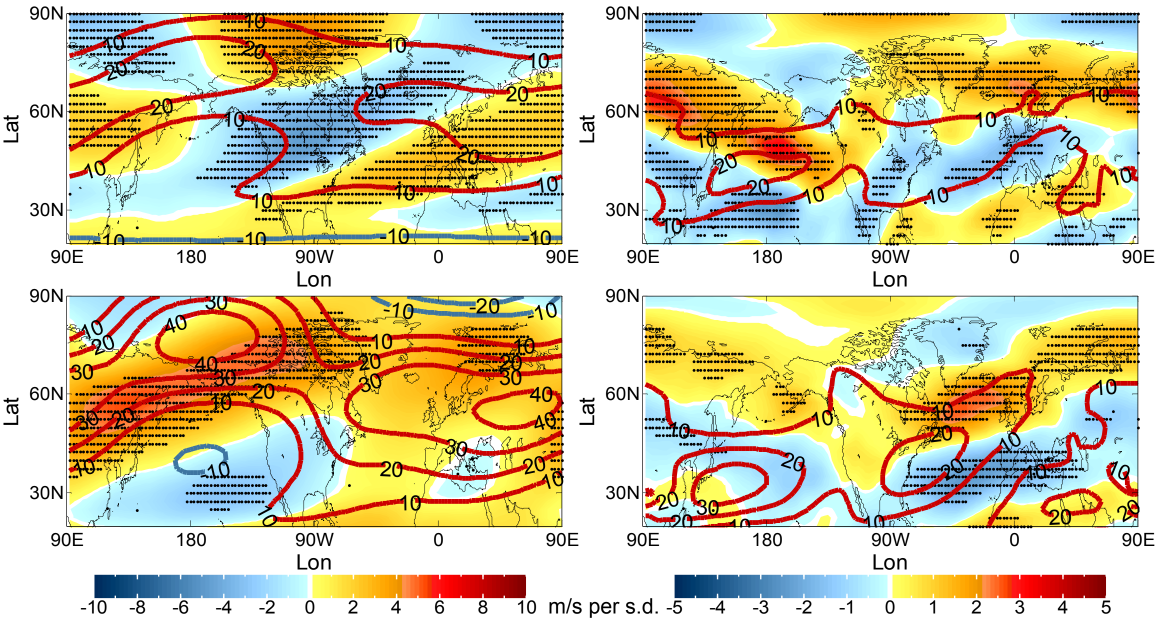

Supplementary Figure S6. October (top) and DJF-mean (bottom) zonal wind regressed on October Z70hPa PC 2 on 10hPa (left) and 500hPa (right) pressure levels (shaded), compared with climatological mean states (contours with an interval of 10 m/s; positive/negative values in red/blue, respectively). The black dots indicate the 95% confidence interval of the regression based on a two-tailed *t*-test. The maps were generated by MathWorks MATLAB R2013b with M_Map (http://www.eos.ubc.ca/~rich/map.html).

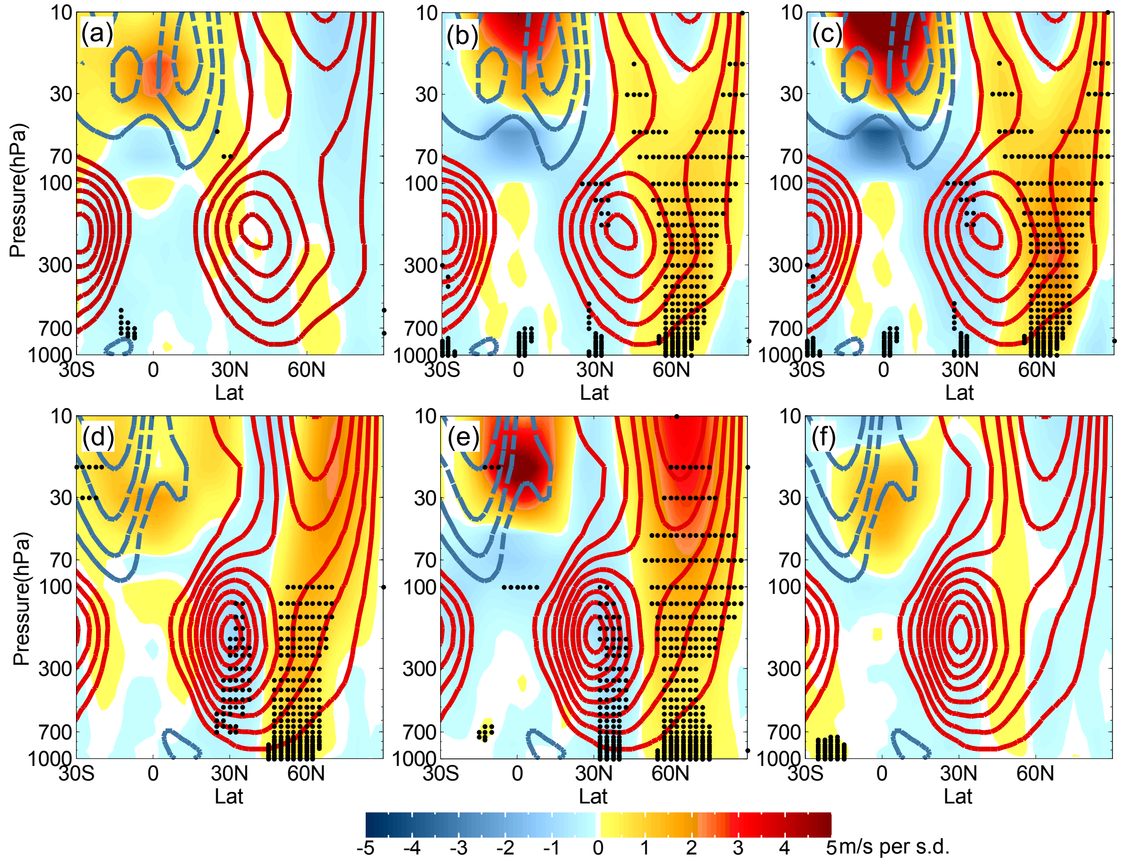

Supplementary Figure S7. Same as Fig. 4 but for October zonal mean zonal wind regressed on (a) October SIC PC1, (b) October Z70hPa PC2, and (c) September SST PC3. (d-f) Same as (a-c) but for the DJF-mean zonal mean zonal wind.

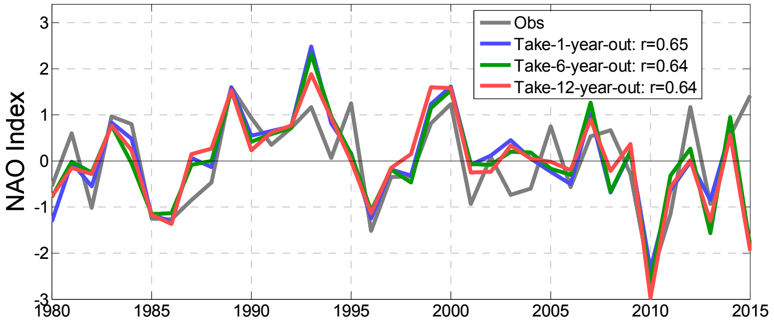

Supplementary Figure S8. Same as Fig.1a but for cross-validated forecast of NAO index with October Z70hPa PC2 replaced by SAI.
